# Chiari Syndrome: Advances in Epidemiology and Pathogenesis: A Systematic Review

**DOI:** 10.3390/jcm12206694

**Published:** 2023-10-23

**Authors:** Raquel Rodríguez-Blanque, Cristina Almazán-Soto, Beatriz Piqueras-Sola, Juan Carlos Sánchez-García, Andrés Reinoso-Cobo, María José Menor-Rodríguez, Jonathan Cortés-Martín

**Affiliations:** 1 Research Group CTS1068, Andalusia Research Plan, Junta de Andalucía, 18071 Granada, Spain; rarobladoc@ugr.es (R.R.-B.); bpiquerassola@gmail.com (B.P.-S.); jcortesmartin@ugr.es (J.C.-M.); 2 San Cecilio University Hospital, 18071 Granada, Spain; 3 Nursing Department, Faculty of Health Sciences, University of Granada, 18071 Granada, Spain; 4 Hospital Vall D’Hebron, 08035 Barcelona, Spain; cristina.almazan@vallhebron.cat; 5 Hospital University Virgen de las Nieves, 18014 Granada, Spain; 6 Department of Nursing and Podiatry, Faculty of Health Sciences, University of Malaga, Arquitecto Francisco Peñalosa 3, Ampliación de Campus de Teatinos, 29071 Malaga, Spain; andreicob@uma.es; 7 Área Sanitaria Santiago de Compostela-Barbanza, Subdirección de Humanización y Atención a la Ciudadanía, 15706 Santiago de Compostela, Spain; mariajosemenor@hotmail.com

**Keywords:** Arnold Chiari Syndrome, Arnold Chiari malformation, posterior cranial fossa, syringomyelia, hydrocephalus, scoliosis

## Abstract

Arnold Chiari syndrome is a rare congenital disease of unknown prevalence and whose origin is still under study. It is encompassed within the posterior cranial malformations, showing a wide spectrum of symptomatology that can range from severe headache, dizziness, and paresthesia to complete asymptomatology. It is for this reason that early diagnosis of the disease is difficult, and it is usually diagnosed in adolescence. Treatment is based on remodeling and decompression of the malformed posterior cranial fossa, although the risk of residual symptoms after surgery is high. The aim of this review is to update all the existing information on this pathology by means of an exhaustive analysis covering all the scientific literature produced in the last 5 years. In addition, it has been carried out following the PRISMA model and registered in PROSPERO with code CRD42023394490. One of the main conclusions based on the results obtained in this review is that the origin of the syndrome could have a genetic basis and that the treatment of choice is the decompression of the posterior cerebral fossa.

## 1. Introduction

Arnold Chiari Syndrome, a rare yet striking condition, lurks in the shadows of medicine, defying understanding and presenting a challenge that few dare to tackle. In a world where rare diseases are often overlooked, this syndrome emerges as a vivid reminder of the complexity of human biology. In its struggle for recognition and understanding, Arnold Chiari Syndrome urges us to look beyond the obvious and seek solutions for those living with this mysterious and challenging condition. In its rarity, we find a valuable lesson about the diversity of the human experience and the need to support research and awareness in the field of rare diseases.

Arnold Chiari syndrome, also known as Arnold Chiari malformation (CM), was first defined in 1890 by the Austrian pathologist Hans Chiari. It is a group of congenital malformations of the central nervous system (CNS), specifically of the posterior cranial fossa and hindbrain [1].

In this syndrome, there are complex complications that can range from herniation of the cerebellar tonsil into the spinal canal through the foramen magnum to a complete absence of the cerebellum. In addition, they may or may not be accompanied by other associated intracranial or extracranial defects, such as syringomyelia (the most common), hydrocephalus, encephalocele, or spinal dysraphism [2].

Depending on the severity of the anatomical defects produced, four types can be differentiated [2]:

- Arnold Chiari malformation type I (CM-I): this is the most common. There is a descent of one or both cerebellar tonsils 5 mm or more below the foramen magnum. It is usually accompanied by syringomyelia.
- Arnold Chiari malformation type II (CM-II) presents with herniation of the brainstem, and the cerebellum is impotent. It is usually accompanied by spinal dysraphism/myelomeningocele.
- Arnold Chiari malformation type III (CM-III): the entire hindbrain is herniated (with or without brainstem) within a cervical meningoencephalocele.
- Arnold Chiari malformation type IV (CM-IV): severe cerebellar hypoplasia.

The origin of this disease is congenital, given by a basioccipital hypoplasia that causes overpopulation of the posterior fossa and, therefore, herniation of the tissues [3]. The true cause leading to this pathogenesis is not yet known, although its genetic basis is being studied in depth, and it has also been shown that it can be acquired after processes such as infections or tumors of the posterior fossa [4].

The compression of the brain structures is responsible for the symptomatology suffered by these patients. Some patients may be asymptomatic; however, the most common symptom among them is an occipital headache associated with Valsalva maneuvers. In addition, they may suffer from many other symptoms, including vertigo, paresthesia, balance problems, muscle weakness, and blurred vision [5].

The prevalence of this disease is unknown [6]. Of the different types, CM-I is the most common, and its prevalence is estimated to be around 1 in 1000 births. In terms of gender, there is no significant difference, the ratio being 1.3 to 1 in favor of females. It is important to bear in mind that, as there are asymptomatic patients, this prevalence may be underestimated [5].

This malformation has been described over the years mainly in adults due to the delay in the onset of its symptoms, but thanks to the introduction of magnetic resonance imaging (MRI), its diagnosis is increasingly frequent in the pediatric population. Therefore, MRI is, at present, the best way to diagnose this disease [5].

The most widely accepted treatment is based on decompression of the posterior cranial fossa by surgical intervention, based on an occipital craniectomy [7]. However, although it is true that it improves the pathophysiology of the disease, it does not guarantee the resolution of the symptoms suffered. These residual symptoms may be palliated pharmacologically by means of NSAIDs or muscle relaxants [5].

From the nursing point of view, one could contribute to the improvement of these patients by trying to alleviate these residual symptoms with alternative therapies, such as exercise therapy, to strengthen the cervical musculature and give greater stability to this area. If the symptoms do not subside or affect the patients’ independence, nursing professionals could also have a beneficial influence in caring for their needs and improving their quality of life [8].

The unification of existing information on Arnold Chiari Syndrome is considered important because, being a rare disease of unknown prevalence, it is difficult to find contributions about it, and the little information that exists is too specific and scattered.

Therefore, the aim of this article is to update the existing information on Arnold Chiari Syndrome in a single report in order to facilitate the search by health professionals, which in turn will benefit the care provided to patients.

## 2. Materials and Methods

The methodology used to carry out this report was a systematic review of the scientific literature published on Arnold Chiari Syndrome. It was carried out following the Preferred Reporting Items for Systematic Reviews and Meta-Analyses (PRISMA) review protocol, which consists of a series of 27-point checks on the most characteristic parts contained in an original article, as well as the process of elaboration of its guidelines.

PRISMA is an essential guideline used in conducting systematic reviews and meta-analyses in the field of scientific research and healthcare. This guideline provides a robust and detailed framework for planning, conducting, and transparently communicating the results of a systematic review. PRISMA helps researchers establish clear guidelines for study selection, data extraction, quality assessment of evidence, and the presentation of results in a uniform and comprehensive manner. By following PRISMA guidelines, it ensures that systematic reviews are more rigorous and comparable, thereby facilitating evidence-based decision-making and the advancement of scientific knowledge.

This systematic review has been carried out following a protocol that can be found on the web page http://www.crd.york.ac.uk/PROSPERO/ (accessed on 19 October 2023) and whose registration number is CRD42023394490.

### 2.1. Eligibility Criteria

The inclusion and exclusion criteria selected for this systematic review were as follows. Articles published between 2018 and 2023, i.e., within the last 5 years, were included. Articles that followed the design of a systematic review and did not have access to the full text were excluded. No restriction on the language of publication was applied.

### 2.2. Sources of Information

The bibliographic search was carried out in the databases of PubMed, Scopus, Cochrane Library, CINAHL, and SciELO. In addition, a manual search was carried out using the list of study references to find other studies that could be relevant to our study.

The language used was obtained using MeSH terms and Health Sciences (DeCS) descriptors. The following descriptors were used: “Arnold-Chiari”, “syndrome”, “malformation”, and “rare disease” with the Boolean operators “OR” and “AND”.

### 2.3. Search Strategy

A table (Table 1) is presented here showing the search strategy used for this work, together with the date on which the search was carried out.

### 2.4. Data Extraction Process

After carrying out the search strategy shown above, the selected articles were transferred to the Mendeley web application using the Mendeley web importer tool. The next step was organization by folders, according to the database from which they were extracted, and all duplicate articles were eliminated.

The studies that were finally included were research articles, original articles, books, and clinical cases that aimed to provide new information on Arnold Chiari Syndrome. The reviewers (C.A.-S.) and (J.C.-M.) independently examined the title, abstract, and keywords of each study and applied the inclusion and exclusion criteria. For studies that were potentially eligible, the same procedure was applied.

Data on quality, patient characteristics, interventions, and outcomes of particular relevance were obtained by the reviewers after a full reading of the articles.

### 2.5. Data Collection Process and Data Collected

The reviewer extracted the following data from each of the articles: authors, year of publication, title, main objective, and the most relevant information about the results obtained in each of them.

In the results section, a more extensive and detailed explanation of the article selection process and the information used for the development of this work is given.

## 3. Results

**Figure 1:**
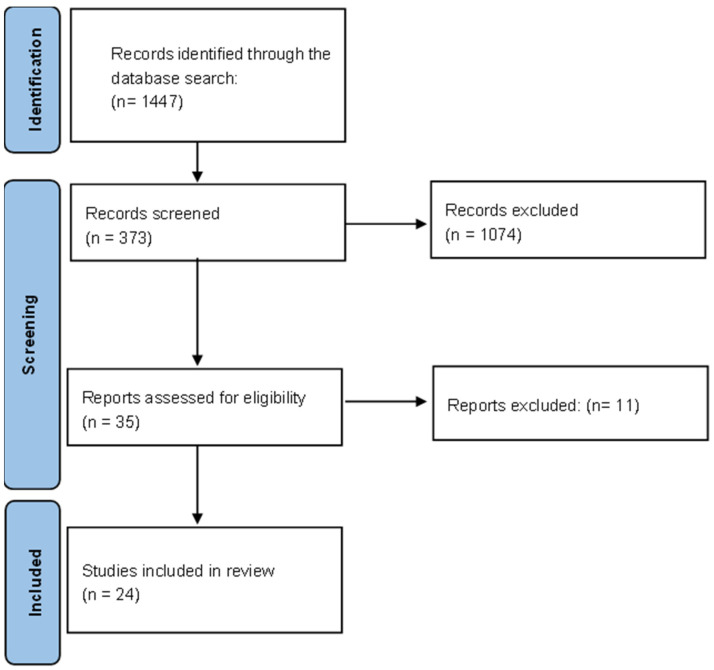
Flow diagram.

The search strategy led to the identification of 1447 articles in the databases, after which the screening process was initiated. Applying the inclusion and exclusion criteria mentioned above, the number of records was reduced to 373 articles. After a title and abstract reading, 35 articles were selected. After this screening, duplicate articles were removed, leaving a total of 24 articles for this systematic review. The flow chart showing the process in schematic form is presented below (Figure 1).

After selecting and reading the articles, the essential information was obtained from each of them and unified in a summary table to organize and schematize all the data. In this way, it was easier to visualize the similarities and differences between the articles. The table of results is shown below (Table 2).

## 4. Discussion

The majority of published articles on Arnold Chiari Syndrome (ACS) agree that the prevalence of this condition is unknown and often underestimated; this could be attributed to the significant number of asymptomatic cases it presents. Some authors have tried to determine its prevalence. According to Ciaramitaro et al. [9], the prevalence of CM in Italy is 7.74 CI 95% (6.965–8.596), and its incidence is 3.08 CI 95% (2.605–3.635). They also studied the incidence and prevalence of syringomyelia due to the high association with this syndrome, resulting in a prevalence of 4.84 CI 95% (4.124–5.527) and an incidence of 0.82 CI 95% (0.599–1.137).

Over the past five years, several studies have attempted to demonstrate the genetic basis of this disease. Authors such as Provenzano et al. [10], Sadler et al. [11], and Musolf et al. [12] investigated exome sequencing in CM-I patients. All of them observed rare genetic variants in these patients, each focusing on a different part of the genetic information. Provenzano et al. [10] state that there are nonsense genetic variants or variants leading to truncated proteins in chromatin remodeling genes, which would result in malformation. In the same line, Sadler et al. [11] observed rare and de novo variants in chromodomain genes, which are closely related to chromatin. They particularly highlighted rare variants transmitted in CHD3 and de novo loss of function in CHD8; this was associated with larger head circumference, macrocephaly, and the characteristic posterior brain displacement of CM-I in their patients. On the other hand, when Musolf et al. [12] studied exome sequencing, they focused on new haplotypes (1q43-44 and 12q23) that appeared in CM-I cases, encompassing significant genomic regions with a considerable number of candidate genes.

Other authors like Urbizu et al. [13] and Martínez-Gil et al. [14] studied different types of genes. Urbizu et al. [13] observed rare variants in collagen genes and linked them to the presence of connective tissue-related symptoms in CM-I patients. However, Martínez-Gil et al. [14] found rare variants in genes regulating bone mineral density (BMD) associated with craniofacial development but could not establish a direct correlation between BMD and CM-I, warranting further study.

In this period of time, Aydin et al. [15] and Heffez et al. [16] have studied some pathophysiological aspects of this disease. Aydin et al. [15] observed increased cerebrospinal fluid (CSF) and lateral ventricle in CM-I cases, supporting the hydrocephalus suffered by these patients. Similarly, Heffez et al. [16] confirmed a relationship between the extent of tonsillar ectopia and the probability of suffering many of the symptoms, but not related to their severity.

Authors such as Lázaro et al. [17] and Ciaramitaro et al. [18] tell us about the characteristic symptomatology of CM. Lázaro et al. [17] studied the verbal fluency of patients with CM-I compared to a healthy control group and found that scores were significantly lower in the CM-I group. In addition, Ciaramitaro et al. [18] studied migraine associated with CM-I and the clinical demographic characteristics of the patients.

Over the years, CM has been associated with many other pathologies. Specifically, as of 2018, Di Rocco et al. [19], Eppelheimer et al. [20], Pan et al. [21], and Turgut et al. [22] have studied the correlation of CM-I to other types of syndromes.

Regarding treatment, four authors have studied the optimal approach in recent years. Among them are García et al. [23], Menezes et al. [24], Rangari et al. [25], and Spena et al. [26]. All of them agree that surgical management with posterior fossa decompression is the best treatment option to improve physiopathology.

There are numerous published clinical cases that demonstrate the validity of all this information. Both Goel et al. [27] and Florou et al. [28] discuss real cases in the year 2020. Goel et al. [27] highlight in their clinical case that the neurological symptoms of a patient with CM worsened following a tethered cord surgery.

The primary limitation of this article lies in the lack of knowledge surrounding Arnold Chiari Syndrome. Being a rare condition with an unknown prevalence, there are limited studies that provide comprehensive information, and existing studies often contain highly specific and scattered information.

To contribute to new lines of research on this condition, it is suggested to continue advancing genetic studies to specify the genes involved and raise awareness about this condition wherever possible. Additionally, basic nursing care can be applied, much like with any severely dependent patient who has lost significant functionality, but no specific care plan exists for patients with this syndrome. The focus could be directed towards cervical care due to discomfort and potential neck immobility, as well as addressing possible pressure injuries, particularly at the occipital level. If mobility issues arise, a tailored exercise plan adapted to these patients would be beneficial. In conclusion, this topic must continue to be studied, both in terms of the origins of the condition and the nursing care plans that should be applied.

## 5. Conclusions

At the end of this work, it has been shown that the information available on this malformation is limited and very specific. This article can be very valuable, as it compiles and updates all the existing knowledge on this pathology in the last 5 years. Healthcare professionals can rely on this article to resolve uncertainties and provide more appropriate healthcare to this type of patient. It reiterates the importance of genetic studies on this disease and the need for personalized nursing care plans for these patients for the advancement of this pathology.

## Figures and Tables

**Table 1:** Search strategy.

Database	Search Strategy
PubMed	((“arnold chiari malformation”[MeSH Terms] OR (“arnold chiari”[All Fields] AND “malformation”[All Fields]) OR “arnold chiari malformation”[All Fields] OR (“arnold”[All Fields] AND “chiari”[All Fields] AND “syndrome”[All Fields]) OR “arnold chiari syndrome”[All Fields]) AND (“rare diseases”[MeSH Terms] OR (“rare”[All Fields] AND “diseases”[All Fields]) OR “rare diseases”[All Fields] OR (“rare”[All Fields] AND “disease”[All Fields]) OR “rare disease”[All Fields])) AND (2018:2023[pdat]) NOT (review)
Scopus	(Arnold AND Chiari AND syndrome) AND (rare AND disease) AND (LIMIT-TO (PUBYEAR, 2023) OR LIMIT-TO (PUBYEAR, 2022) OR LIMIT-TO (PUBYEAR, 2021) OR LIMIT-TO (PUBYEAR, 2020) OR LIMIT-TO (PUBYEAR, 2019) OR LIMIT-TO (PUBYEAR, 2018)) AND (EXCLUDE (DOCTYPE, “re”))
CINAHL	(Arnold Chiari syndrome) AND (rare disease)
Cochrane Library	(Arnold Chiari syndrome) AND (rare disease)
SciELO	(Arnold Chiari syndrome [Todos los indices] and rare disease [Todos los indices])

**Table 2:** Table of results.

Author	Year	Title	Aims	Results
Ciaramitaro et al. [9]	2020	“Syringomyelia and Chiari Syndrome Registry: advances in epidemiology, clinical phenotypes and natural history based on a North Western Italy cohort”.	To estimate the incidence and prevalence of CM and syringomyelia in northeastern northeastern Italy.	The prevalence of syringomyelia was estimated at 4.84 CI 95% (4.124–5.527), while that of Chiari was 7.74 CI 95% (6.965–8.596); incidences were 0.82 CI 95%(0.599–1.137) and 3.08 CI 95% (2.605–3.635), respectively.
Provenzano et al. [10]	2021	“Chiari 1 malformation and exome sequencing in 51 trios: the emerging role of rare missense variants in chromatin-remodeling genes”.	To discover and validate the genes genes associated with CM-I.	It shows that CM-I cases are mainly dependent on variants in chromatin remodeling genes; in most variants in chromatin remodeling genes, in most cases, they are nonsense mutations, and in very rare nonsense mutations, very few of them lead to a truncated protein.
Sadler et al. [11]	2021	“Rare and the novo coding variants in chromodomain genes in Chiari I malformation”.	Identify genes and genetic variants genetic variants associated with CM-I risk.	Significant enrichment of rare non-synonymous variants and de novo variants in chromodomain genes was observed in patients with CM-I, including three de novo loss-of-function variants in CHD8 and a notable burden of rare, transmitted variants in CHD3. Overall, individuals with CM-I exhibited larger head circumference, with many carrying rare CHD variants associated with macrocephaly. Haploinsufficiency for CHD8 resulted in macrocephaly and posterior brain displacement reminiscent of CM-I; this highlights the involvement of chromodomain genes and excessive brain growth in the pathogenesis of CM-I.
Musolf et al. [12]	2019	“Small posterior fossa in Chiari I malformation affected families is significantly linked to 1q43-44 and 12q23-24.11 using whole exome sequencing”.	Demonstrate the relationship between the measurement of the posterior fossa and sequencing of the exome sequencing in patients with CM-I.	Two novel linked haplotypes are identified at 1q43-44 and 12q23 for small posterior fossa, one of the underlying causes of CM-I. Both haplotypes encompassed large genomic regions, thereby incorporating a substantial number of candidate genes.
Urbizu et al. [13]	2021	“Rare functional genetic variants in COL7A1, COL6A5, COL1A2 and COL5A2 frequently occur in Chiari Malformation Type 1”.	Identify genetic risk variants of CM-I.	Rare variants in collagen genes were observed to be common in CM-I. Some of these genes have previously been associated with musculoskeletal phenotypes but never with this pathology until now. These findings underscore the contribution of rare genetic variants in these genes to CM-I and the association between the presence of connective tissue-related symptoms in CM-I patients and the occurrence of such variants.
Martínez-Gil et al. [14]	2022	“On the association between Chiari malformation type 1, bone mineral density and bone related genes”.	Identifying rare variants in genes associated with bone mineral density (BMD) and their association with CM-I.	Genetic variants have been discovered in genes related to bone mineral density (BMD) in certain patients with CM-I. It has been observed that these variants contribute to craniofacial development in various ways or have been previously associated with the condition. Further investigation is warranted to better understand the relationship between bone-related genes and CM-I and to gather more evidence of their contribution to the etiology. A direct correlation between bone mineral density and CM-I was not observed.
Aydin et al. [15]	2019	“Comparative Volumetric Analysis of the Brain and Cerebrospinal Fluid in Chiari Type I Malformation Patients: A Morphological Study”.	Researching the differences in brain volume between CM-I patients and a healthy population revealed notable findings.	An increase in cerebrospinal fluid (CSF) and lateral ventricle volume was observed in CM-I patients compared to the control group, supporting ventricular enlargement and hydrocephalus in these patients. However, the volume of the brain and cerebellum was reduced. While there were no significant differences in white matter volumes, gray matter was significantly lower in CM-I patients. This reduction in gray matter might underlie certain cortical dysfunctions seen in individuals with this condition.
Heffez et al. [16]	2020	“Is there a relationship between the extent of tonsillar ectopia and the severity of the clinical Chiari syndrome?”.	Investigating the correlation between the extent of tonsillar ectopia and the prevalence and severity of symptoms associated with CM.	Regression analysis confirmed a connection between the extent of tonsillar ectopia and the likelihood of experiencing many symptoms. However, the severity of symptoms does not directly correlate with the extent of tonsillar ectopia.
Lázaro et al. [17]	2018	“Chiari Type I Malformation Associated With Verbal Fluency Impairment”.	Investigating if verbal fluency is affected in CM-I patients.	Significantly lower scores were found for the CM-I group in terms of verbal fluency compared to the control group. After controlling for tendencies of anxious and depressive symptoms, it was observed that verbal fluency could not be predicted by these variables. Therefore, it can be concluded that individuals with CM-I exhibit reduced verbal fluency, and this difference is not attributed to depression or anxiety.
Ciaramitaro et al. [18]	2022	“Migraine in Chiari 1 Malformation: a cross-sectional, single centre study”.	Examine the prevalence of migraine in CM-I patients and compare the clinical-demographic characteristics with CM-I without migraine.	Out of 230 patients, 78 exhibited migraine headaches, with 44 out of those 78 experiencing aura; 0.5387 CI 95% (0.4487–0.6287). Among these, 58 had comorbid migraines attributed to CM-I; 0.1357 CI 9% (0.0779–0.1935). The prevalence of migraine was higher in isolated CM-I patients compared to the remaining subjects. While migraine was more prevalent in females, age and gender were not risk factors for its occurrence; however, it was associated with the isolated CM-I phenotype. Thus, a high prevalence of migraine in CM-I patients is demonstrated, with a significant association between migraine and isolated CM-I.
Di Rocco et al. [19]	2023	“Surgical management of Chiari malformation type 1 associated with MCAP syndrome and study of cerebellar and adjacent tissues for PIK3CA mosaicism”.	Propose a potential relationship between the PIK3CA gene and the cerebral phenotype in patients with megalencephaly-capillary malformation polygyria (MCAP) and CM-I.	Both in CM-I and MCAP, alterations in cerebrospinal fluid (CSF) dynamics were observed, specifically syringomyelia and hydrocephalus, which necessitated surgical intervention. Targeted sequencing of PIK3CA determined the variable allelic frequency of the postzygotic variant in both the cerebellum and adjacent bone/connective tissues.
Eppelheimer et al. [20]	2018	“A Retrospective 2D Morphometric Analysis of Adult Female Chiari Type I Patients with Commonly Reported and Related Conditions”.	Identify the morphological characteristics of conditions associated with CM-I and explain the inconsistent results of comparisons between cases and controls.	A reduced McRae line length was observed in CM-I participants with syringomyelia. On the other hand, tonsillar position was decreased in CM-I participants with Ehlers–Danlos syndrome, and the basion to axial line posterior distance was greater in CM-I participants with scoliosis. Furthermore, differences were found between CM-I participants and healthy controls regardless of associated conditions, indicating that the prevalence of these conditions is not strongly linked to CM-I. The inconsistent findings in the radiographic literature are not explained by these conditions in the CM-I samples.
Pan et al. [21]	2018	“Chiari I Malformation and Basilar Invagination in Fibrous Dysplasia: Prevalence, Mechanisms, and Clinical Implications”.	Determine the prevalence and risk factors of CM-I and basilar invagination (BI) in patients with McCune Albright syndrome (MAS).	Craniomorphometric and volumetric analyses in patients with MAS identified cranial constriction and settling as the primary mechanisms for anomalies at the skull base, with intracranial hypertension playing a lesser role. Furthermore, there was a noted progression of odontoid changes with age, but not in the tonsillar position. Endocrinopathies associated with BI in MAS included precocious puberty, hyperthyroidism, and hypophosphatemia, but they were not linked to CM-I. Scoliosis was correlated with both CM-I and BI.
Turgut et al. [22]	2020	“Chiari I Malformation and Craniosynostosis”.	Highlight the existing association between CM-I and craniosynostosis.	CM-I is commonly associated with syndromic multisutural craniosynostosis, characterized by early fusion of the lambdoid sutures and synchondrosis of the skull base. The standard treatment for these conditions involves simultaneous or sequential remodeling of the cranial vault and suboccipital decompression. However, it is widely accepted to perform cranial vault expansion or remodeling procedures before the decompression.
García et al. [23]	2018	“Comparison between decompressed and non-decompressed Chiari T Malformation type I patients: A neuropsychological study”.	Examine whether posterior fossa decompression leads to an improvement in the cognitive profile deficit in CM-I patients.	CM-I patients exhibit lower cognitive performance in executive function, verbal fluency, spatial cognition, language, processing speed, verbal memory, facial recognition, and theory of mind compared to patients in the control group. These results persist even after statistically controlling for physical pain and anxious–depressive symptoms. These findings underscore a cognitive deficit associated with CM-I, independent of posterior fossa decompression surgery.
Menezes et al. [24]	2018	“Syringobulbia in pediatric patients with Chiari malformation type I”.	Enhancing the understanding of the presentation, treatment, and surgical outcomes of CM-I.	The incidence of syringobulbia in CM-I patients was 4%, and all of them had concurrent syringomyelia. The syringobulbia involved the medulla in all cases and communicated with the fourth ventricle in 54% of cases. Posterior fossa decompression with intradural exploration and duraplasty has proven effective for these patients.
Rangari et al. [25]	2021	“Type I Chiari Malformation Without Concomitant Bony Instability: Assessment of Different Surgical Procedures and Outcomes in 73 patients”.	Compare different treatment strategies for CM-I using objective outcome measures.	Patients with minimal symptoms underwent posterior fossa bony decompression (PFBD), while those with more severe symptoms received both bony and dural decompression (PFBDD) of the posterior fossa. The PFBDD group predicted a poor short-term postoperative outcome. Findings indicate that PFBD appears to be a durable procedure, whereas the PFBDD group experiences complications and late deterioration. Posterior fossa fixation does not yield better long-term outcomes compared to decompression alone.
Spena et al. [26]	2020	“Management of Chiari Malformation”.	Provide detailed information about the management of CM-I.	Surgical management is the preferred treatment for this condition. However, there are still many patients who do not experience symptom relief after surgery. There are surgical nuances that are still under debate, but it appears that the issue lies in understanding the subtle differences between patients. One possible solution would be to adopt a more comprehensive approach that does not solely focus on the skull base and posterior fossa.
Goel et al. [27]	2020	“Tethered cord and Chiari formation: Analysis of treatment in a relatively rare clinical situation”.	Contributing additional information to the literature on Chiari Malformation by explaining a real-life case.	Following surgery for a tethered spinal cord, the patient’s neurological symptoms continued to worsen, leading to the development of spastic tetraparesis, urinary retention, and constipation. An atlantoaxial fixation procedure was performed, resulting in improved limb function and urinary and bowel control. The presence of symptomatic Chiari in conjunction with a tethered cord is a rare clinical event. Surgical treatment of Chiari formation can lead to a rewarding clinical recovery.
Chrysoula et al. [28]	2020	“Acute Visual Loss Secondary to Arnold Chiari Type I Malformation Completely Resolving After Decompressive Posterior Fossa Surgery”.	Illustrating a real case of CM-I.	Consider the case of a 22-year-old young woman who presented with sudden unilateral visual loss following hearing loss. MRI revealed a small, almost subclinical herniation of the cerebellar tonsils. The patient underwent occipital craniotomy with posterior fossa decompression, resulting in a favorable outcome with papilledema regression and full recovery of visual acuity. The occurrence of papilledema associated with CM-I is rare.
Florefice et al. [29]	2022	“Pseudo Chiari with holocord syringomyelia secondary to cerebrospinal fluid hypotension. Case report”.	Contributing novel information about Pseudo Chiari with the presentation of a clinical case.	After surgically decompressing the posterior fossa using an epidural blood patch and a dural patch, it was observed that the patient achieved favorable clinical outcomes.
Gabr et al. [30]	2022	“Chiari Type III: Experience of Outcome for 15 Cases”.	Highlight the key findings from 15 cases of CM-III.	Initially, eight patients required ventriculoperitoneal shunting, while the other seven patients began with a normal delivery. Subsequently, six more patients needed such shunting due to cerebrospinal fluid leakage and increased intracranial pressure. Only four patients required a blood transfusion. Therefore, it is affirmed that there are variations in outcomes, and not all CM-III cases will result in death or severe developmental delay. Proper management leads to a favorable prognosis for the condition.
Deleu et al. [31]	2022	“Brain overgrowth associated with megalencephaly-capillary malformation syndrome causing progressive Chiari and syringomyelia”.	Gather information on a case involving the association of megalencephaly-capillary malformation (M-CM) with CM-I and syringomyelia.	The case involves an infant with M-CM who developed progressive CM-I and syringomyelia, reflecting disproportionate growth of the cerebellum/posterior fossa; this justified a suboccipital craniectomy and C1 laminectomy with duraplasty. At three, six-, and nine-months post-surgery, the only residual deficit in the patient was mild gait disturbance.
Chu et al. [32]	2022	“Neck pain and Headache Complicated by Persistent Syringomyelia After Foramen Magnum Decompression for Chiari I Malformation: Improvement with Multimodal Chiropractic Therapies”.	Present information about a real case of CM-I treated with foramen magnum decompression.	This case features a patient with neck pain, headache, and persistent syringomyelia following CM-I decompression. The symptoms improved after multimodal rehabilitation and chiropractic techniques. Due to limited and low-level evidence for these interventions in patients with syringomyelia and persistent symptoms post-decompression, these therapies cannot be broadly recommended. However, they might be considered on a case-by-case basis.

## Data Availability

Data are available on request from the corresponding author.
